# The status of health economic evaluation within decision making in Austria

**DOI:** 10.1007/s10354-019-0689-8

**Published:** 2019-03-12

**Authors:** Ingrid Zechmeister-Koss, Michal Stanak, Sarah Wolf

**Affiliations:** 1 Ludwig Boltzmann Institute for Health Technology Assessment, Garnisongasse 7/20, 1090 Vienna, Austria; 2 Department of Philosophy, University of Vienna, Vienna, Austria

**Keywords:** Economics, Cost-effectiveness analysis, Cost-utility analysis, Efficiency, Reimbursement, Ökonomie, Kosten-Effektivitäts-Analyse, Kosten-Nutzwert-Analyse, Effizienz, Vergütung

## Abstract

**Background:**

Given limited resources compared to the demand for them, spending resources efficiently is important. Key methods applied for supporting efficient resource allocation are health economic evaluations.

**Methods:**

Based on secondary literature, we analyze international challenges for using two types of economic evaluations—cost-effectiveness analysis and cost-utility analysis—in reimbursement decisions and reflect on them for the Austrian case.

**Results:**

The main challenges with the application of economic evaluations are related to the methods, the decision-making culture, and the respective system. The challenges also apply to the Austrian Bismarck system, where almost no formal requirements for using economic evaluations exist, except on a case-by-case basis. Resource allocation in Austria hence occurs, for the most part, implicitly.

**Conclusion:**

One way forward towards more explicit efficiency considerations may be to consider more descriptive study types and foster capacity building, standardization of methods and presentation of results, and a mandatory detailed guideline.

## Zusammenfassung

**Grundlagen:**

Angesichts begrenzter Ressourcen im Vergleich zur Nachfrage sind Fragen einer effizienten Verwendung der Mittel unumgänglich. Methoden, die eine effiziente Allokation unterstützen, sind gesundheitsökonomische Evaluationen.

**Methodik:**

Es werden die internationalen Herausforderungen für die Anwendung von Kosten-Effektivitäts- und Kosten-Nutzwert-Analysen in Refundierungsentscheidungen auf Basis von Sekundärliteratur analysiert und für Österreich systematisch reflektiert.

**Ergebnisse:**

Wesentliche Herausforderungen bei der Berücksichtigung gesundheitsökonomischer Evaluationen bilden methodische Limitationen, die Entscheidungskultur und die Charakteristika des jeweiligen Gesundheitssystems. Diese Herausforderungen gelten insbesondere für das österreichische Bismarck-System, in dem kaum formelle Anforderungen für die Verwendung gesundheitsökonomischer Evaluationen existieren. Die Ressourcenallokation erfolgt daher in Österreich weitgehend implizit.

**Schlussfolgerung:**

Eine Weiterentwicklung zu mehr expliziter Berücksichtigung von Effizienzfragen könnte durch Anwendung von Evaluationstypen mit einem stärker beschreibenden Charakter, einschlägige Aus- und Weiterbildung, Standardisierung von Methoden, transparente Präsentation von Ergebnissen sowie durch eine verbindliche detaillierte methodische Leitlinie für Hersteller gelingen.

## Introduction

Similar to other advanced health care systems around the world, the Austrian health care system faces major challenges in order to maintain universal coverage [1]. Accelerated demand for health care services related to demographic change and technological developments have increased the pressure on health care budgets and have threatened sustainability of the universal health system (e. g., [2]). Since health care budgets are restricted, every time a new technology enters the health care market, from an economic point of view, resources required to deliver a new intervention must be found by replacing, disinvesting from, (or not investing in) other interventions elsewhere. While some individuals may benefit from the new technology, (silent) displacement may inevitably result in health decrements for others. On top of the disinvestment challenge, health care systems are also confronted with the fact that 20–40% of resources are wasted, meaning that they are spent on ineffective or unnecessary interventions (e. g., duplication of diagnostic tests) [1]. The more inefficiently limited health care resources are used, the more likely it is that patients who would have received treatment if resources had been better used will be denied treatments and health improvements. Moreover, inefficiency in the health system may divert resources from other sectors of the economy such as education or long-term care, where the resources could be used productively [3]. Given the limited resources compared to the demand for them, rationing—in the sense of withholding health care on economic grounds—is de facto unavoidable [4].

As a consequence, efficiency in health care—using resources to get the best value for money [3]—has become increasingly relevant within health policy. While efficiency can be addressed in many forms (e. g., measuring hospital performance or comparing treatment alternatives) and at different levels of the health care system (e. g., choice for treatment at the micro-level or resource allocation between primary and secondary care at the macro-level), the issue of value for money has become a topic, particularly in public reimbursement decisions on benefit coverage (e. g., drugs). To facilitate efficient resource allocation, different methods of health economic analysis have been developed to be integrated in reimbursement processes. Key methods applied are health economic evaluations.

This article will address currently used core methods of economic evaluation—cost-effectiveness (CEA) and cost-utility analysis (CUA)—and describe the extent to which they are applied in assessment, pricing, and reimbursement processes internationally and in Austria. Based on the international evidence on essential challenges for using economic evaluations, the Austrian case will be critically reflected and a potential way forward for using economic evidence in Austria in decision processes will be presented. The article is based on the premise that economic evidence ought to have some role in the decision-making process, yet the theory it is based on is not fundamentally questioned.

## Materials and methods

While the paper is based on secondary literature overall, the individual sections follow different conceptual approaches. The first section that introduces the methods of economic evaluation (focusing on the key methods of CEA and CUA) describes the core methodological principles based on standard textbooks and published papers on methodological foundations.

The second section (international evidence on the current use of economic evaluations in coverage processes) is based on a recently published information synthesis in which political reports, guidelines, legislative texts, handbooks of ministries, organizations and institutions, as well as journal publications were used to create country profiles [5].

For describing the challenges in using economic evaluations (third section), empirical information on factors that hinder their use have been searched for in journal publications and systematically extracted in a tabular form (table available upon request). Studies that were published before 1995 or that were focused on health care systems in developing countries were excluded. The search for publications was based on a hand search and was continued until an exhausted list of challenges was available and additional studies did not add new types of information. The individual challenges were clustered into groups and each cluster was described.

The description of the Austrian health care system and the current use of economic evaluations (section four) is based on published legal documents and health care system reports. For each type of coverage process where economic evaluations have already been used, a case study of an economic evaluation is presented. It describes the purpose of the study, the method that was applied, the core results, and its role in the decision-making process.

In the fifth section, the use of economic evaluations in Austria is systematically analyzed by comparing the Austrian system and economic study characteristics with the clusters of challenges for applying economic evaluations identified earlier. Based on the results, ideas for a potential way forward are presented.

## Results

### The methods of economic evaluation

Economic evaluation is defined as “the comparative analysis of alternative courses of action in terms of both their costs and consequences” [6, p. 4]. Thus, it establishes the relative costs and impacts of health interventions, with the underlying objective of maximizing population health for the available resources [7]. This includes identifying, measuring, and valuing costs and outcomes of the alternative interventions that are considered within such an analysis. Different types of economic evaluation exist, whereby CEA and CUA (which is sometimes classified as a sub-group of CEA) are used most widely. Both of them measure costs in monetary units, but they differ in the way they measure outcomes. While the former quantifies outcomes in the form of “natural units” (e. g., life years gained, depression-free days reduced), the latter determines the benefit of an intervention in the form of utilities, whereby the term “utility” refers to the “preference individuals or society may have for a particular set of health outcomes” [6, p. 14]. The attraction of utilities has been that they allow inclusion of health-related quality of life issues and at the same time introduce a generic outcome measure. One of the most often used utility measures in economic evaluations are “quality-adjusted life years” (QALYs). In QALYs, length of life and health-related quality of life are consolidated into a single value [8].

**Fig. 1:**
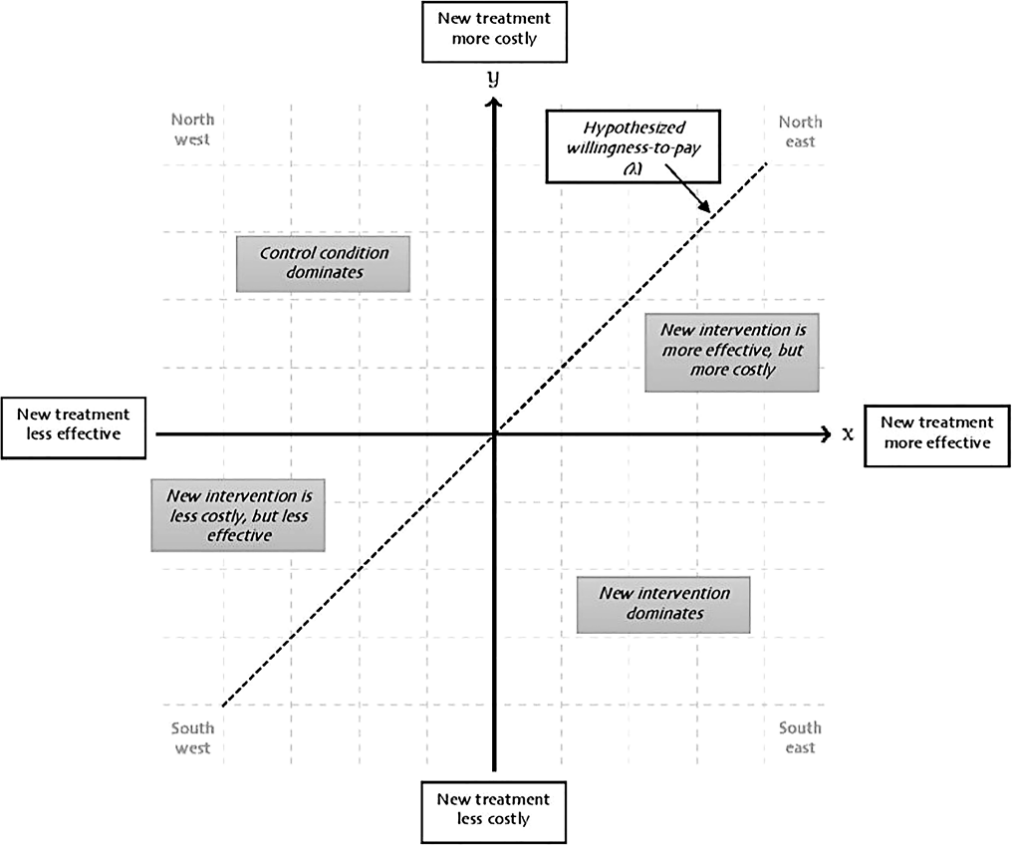
Cost-effectiveness plane, adapted from [6]

The results of a CEA or CUA are described in terms of the ratio of incremental costs per unit of incremental health benefit, the incremental cost-effectiveness ratio (ICER). The results can be classified within a so-called cost-effectiveness plane (Fig. 1). If an intervention is less costly and more effective (south-east quadrant) or, vice versa, if it is more costly and less effective (north-west quadrant), the interpretation of the result is straightforward. The former option suggests adopting the intervention, while in the latter case, it may be rejected. However, most often, the intervention is both, i.e., more effective but also more expensive than the alternatives (north-east quadrant). In this case, a decision rule is required. If the aim is to produce the maximum health within a population, then the criterion for adopting the intervention will be that the intervention evaluated is a better use of scarce resources than spending the money on something else (opportunity costs). To address this, the cost-effectiveness threshold has been developed which is “an estimate of health forgone as other activities are displaced to accommodate the additional costs of new technologies” [9, p. 1]. Therefore, adopting technologies with an ICER below the cost-effectiveness threshold means that the health gains will outweigh the decrements, while it is the other way round if a technology with an ICER above the threshold is reimbursed. This shows that the aim of economic evaluations is not to save money but to gain as much health as possible from the available resources [7]. The underlying utilitarian principle of maximizing health means that CEA and CUA fall within the field of normative economics (as opposed to positive economics) [10]. Thus, they recommend specific policy actions based on the normative judgement that one goal of health policy ought to be maximizing population health [11].

### Application of health economic evaluations in reimbursement decisions internationally

Economic evaluations have been widely applied for informing reimbursement decisions in publicly funded health care systems in Europe, but also in countries outside Europe such as Australia or Canada [3, 12]. The most commonly used types are CEA and CUA [13]. However, the extent to which they are used varies. For example, in the Netherlands (in inpatient care), Sweden, or the United Kingdom, its use is extensive, while in Germany or the United States, it is rather limited [5, 14]. The field in which economic evaluations have been used most extensively is drug reimbursement decisions. The first country that included economic evaluations as a requirement in the submission guidelines for the pharmaceutical industry was Australia. According to Drummond (2013) [12], half of the countries in the European Union request economic analyses to varying degrees (e. g., for high-cost medicines in the Netherlands or France, for drugs with substantial additional clinical benefit in Austria and France [5]). More recently, countries in Latin America and Asia have also expressed interest in using economic evidence more systematically in coverage processes [12].

Considerable differences exist regarding the way such studies are applied in the reimbursement processes. Some jurisdictions (e. g., Italy, Spain)—while overall committing themselves to consider efficiency in their decisions—have not defined formal requirements for the manufacturers on how to conduct economic evaluations. Others (e. g., Scotland, Canada, Belgium) have defined more precise methodological guidelines and procedural arrangements; however, there are still differences concerning the range of technologies (e. g., all drugs or only a selection) that are subject to economic evaluations [12]. Variations also exist regarding the appraisal of the submitted manufacturers’ studies. In some cases this is done by the staff within the government or an HTA agency (Finland, Norway, Sweden), in others by a separate group (e. g., from academia) [5]. Appraisal of the economic evaluations in some countries means conducting an own study and comparing it with the submission (e. g., in England), while in other jurisdictions (e. g., Australia, Canada, the Netherlands) it refers to critically appraising the submitted studies [5]. Finally, differences exist concerning the extent to which the committees that are involved in the decision processes represent expertise in economics, which has implications for the interpretation of the studies and the weight they may have in the decision making itself [12].

Little evidence exists regarding the impact of using economic evaluations in decision-making. It was stated that whether allocation of health care resources has changed or whether health overall has improved as a result of using economic evaluations is difficult to answer, because the counterfactual data (how would decision-making have developed if economic evaluations had not been incorporated) is unknown [12] and too many different factors influence the final decisions [15]. However, economic evaluations require specific types of data which may have contributed to improvements in clinical study design (e. g., more focus on patient-relevant outcomes in clinical trials, more head-to-head trials) and to the development of methods (e. g., network meta-analysis in the absence of head-to-head trials) [12]. Furthermore, economic evaluations may have contributed to targeting interventions to specific sub-populations (identified in economic evaluations) and to an increasing use of managed entry agreements [12]. It was demonstrated that in countries that use economic evaluations, the ICER is a prominent factor in decision making; however, decision makers consider other criteria (clinical need, degree of priority etc.) as well [12, 15]. Applying a transparent decision criterion such as the ICER has resulted in high-cost interventions being more evenly distributed across the country in contrast to “postcode rationing” [12].

### Challenges to the application of economic evaluations

A large number of challenges for using economic evaluations in decision making have been described in the literature. According to their nature, they can be classified into different categories (see Table 1) that are described below.

**Table 1:** Classification of challenges to the application of economic evaluations

Category	Type of challenge
Method-related challenges	Methodology
	Data used within economic evaluations
	Reporting and communication
	Applicability
Decision maker challenges	Lack of knowledge and methodological expertise
	Nature of decision making
	Concerns for negative impact
System-related challenges	Health care system type
	Decision making culture
	Administrative culture

#### Challenges related to the method

##### Methodology

Firstly, methodological challenges in the method of economic evaluation itself have been identified as a reason for its limited use. One commonly raised methodological issue concerns limitations regarding QALYs (e. g., [4, 16]). These include limitations regarding the reliability, precision, accuracy, and validity of measuring well-being with the QALY-approach. For example, while QALYs address health outcomes, they do not capture processes in “health production” such as compassionate health care that may be as valuable to a patient as the actual health outcome. Other concerns are related to unproven empirical assumptions that are inherent in QALYs, for example, that QALY gains to the severely ill have the same value as gains to the less severely ill [16].

##### Data used within economic evaluations

In economic evaluations, many different types of data are needed (e. g., cost data, data on the effectiveness of an intervention, data on the natural history of diseases). A major limitation to using an economic evaluation is if users are concerned with the quality of the underlying clinical evidence that was used in the economic evaluation. The same is true for unreliable cost data or cost data that are not relevant for the jurisdiction in question [17]. Existing data limitations also mean that study results are based on many assumptions, which increases uncertainty and lack of credibility [18].

##### Reporting and communication

The way study results are reported and communicated can be another limitation. For example, studies are often poorly written or report only final aggregated results. The latter has been observed for cost data in particular [17]. In other cases, a lot of emphasis is put on scientific rigor using many technical terms and elaborated statistical tools, while authors neglect communication of the results to the non-scientific community [19].

##### Applicability

Evidence has shown that economic evaluations are sometimes not undertaken in a timely fashion or they focus on very narrow and specific questions not relevant for addressing the complexity of the decision problems [18]. It has also been pointed out that studies are often undertaken by applying a rigid standard framework without fully understanding what the actual needs of the decision makers are [20].

#### Decision maker challenges

##### Lack of knowledge and methodological expertise

If decision makers or those who are responsible for critical appraisal of economic evaluations have little knowledge about the methods, they will find it difficult to use them and to recognize the benefit of using this type of economic evidence in decision making [18, 19]. Insufficient expertise also often leads to misunderstandings and wrong perceptions. For example, the term cost-benefit analysis is often used by decision makers as a term for economic analysis in general, while for economists, it is related to one very specific type of economic evaluation [19, 21].

##### The nature of decision making

Williams and Bryan (2007) [11] have pointed out on a more conceptual level that the idea that economic evidence would be directly applied to a policy problem is unrealistic per se, because it follows a rational model of research utilization that assumes that decision makers are able and willing to act on research findings. However, the policy environment is much more complex and the decision making is subject to multiple influencing factors. The application of economic evaluation requires the decision maker to first have a clear set of objectives/values and second, to consider the maximization of health gains as one of those core objectives/values to pursue. Doubts have been raised on both assumptions [11].

##### Concerns for the negative impact of economic evaluations and cost-effectiveness thresholds

Worries have been raised that health care purchasers may use economic evaluation to reinforce existing beliefs, support pre-determined decisions [22], delay decisions [19], or that the studies may be misused by the industry (e. g., manufacturers price the interventions just up to the threshold, while without economic evaluations the prices could be lower) [12]. Some critiques have rejected the use of certain types of economic evaluations (cost-utility analysis) because they are concerned that the evaluations are too closely associated with the UK health care system that tends to have a bad reputation in their country, and that this could be considered non-acceptable by the public. As put by a German expert: “Respondents were averse to use of QALYs because they did not want their own methodological practices being associated with HTA practices in the UK” [21, p. 276].

#### System-related challenges

##### Health care system type

Economic evaluations, including the idea of a threshold, strongly rest upon the concept of a fixed budget. In contrast to Beveridge health care systems (e. g., UK, Italy, or Spain), in which the budget is set by the parliament for a given year and therefore clearly defined, the budget in social insurance-based Bismarck systems (e. g., Austria or Belgium) is more volatile by nature. This is because it depends on the income of the health insurance funds, which is in turn based on the income of the insured and cannot be predicted precisely. Additionally, the perspective to be applied in economic evaluations is less straightforward in Bismarck systems because private co-payments often play an important role, which challenges the idea of a applying a public health care system perspective in economic evaluations [23].

At the core of CEA and CUA is the explicit efficiency assessment—supporting maximizing population health within limited resources. However, it has been demonstrated that Bismarck systems are characterized by a general lack of an explicit use of efficiency assessment in decision making [14, 21], while positive discrimination between individuals and/or disease areas according to needs or other factors plays a central role in reimbursement decisions [14]. This indicates that the principle of vertical equity (unequal treatment of unequals) may have a higher priority in resource allocation in Bismarck systems than horizontal equality (equal treatment of equals). However, the QALY approach is based on horizontal equity and the risk for systematically discriminating certain patient groups (e. g., elderly and disabled people) has been criticized as one of the core limitations of QALYs among those who refuse to use economic evaluations [21].

##### Decision-making culture

Taking economic evaluations into account in decision making means that some interventions may be explicitly excluded from public funding if the economic evaluation shows that more health is lost elsewhere than would be gained with the new technology, even if an intervention may have demonstrated some clinical benefit. Yet, in some decision making cultures, the discourse is dominated by the notion that “everything for everybody will be made available” [21, p. 276], including a general aversion towards the idea of explicit rationing (meaning withholding effective health care on the basis of costs in a transparent manner) or even rationalization (withholding ineffective treatments) [21].

It has also been observed that decision making in Bismarck systems is much more influenced by various stakeholder groups than in Beveridge systems. This makes the introduction of rational decision rules such as a cost-effectiveness threshold more challenging [23]. In the absence of centralized coverage processes in Bismarck systems, many reimbursement decisions are de facto made at the individual patient level by clinicians based on their vital role in approving treatments. However, it has been shown that many clinicians are neither willing nor do they have the required expertise to apply economic considerations in their decisions [21]. Not least, lack of transparency in the decision-making culture has been described as being another core barrier to the inclusion of (cost-)effectiveness evidence into the decision-making processes [24].

##### Administrative culture

Torbica et al. (2017) [14] built on the work of public administration research and argued that the administrative culture may be of even greater importance than the health care system type. For example, similar to the UK, Italy and Spain have a Beveridge system. However, their Napoleonic administrative tradition slows down the speed with which decision processes may be changed and new criteria (such as cost effectiveness) may be introduced. This is because in a Napoleonic administrative tradition, public officials usually act on a robust legal basis which needs to be in place first, and this likely slows down the development of procedures for Health Technology Assessment (including economic evaluation). This is different to Anglo-American traditions that allow public officials more freedom to implement policies [14].

##### Financing structures

Decision makers are often faced with a lack of budget flexibility when needing to move resources from one sector to another [18]. This is especially the case in those systems where different funding sources exist for different types of services and/or where planning may take place on both national and regional levels. In those systems, the “thinking in silos” dominates the actions of decision makers. Economic evaluations usually address the entire health care system (if done from a health care system perspective) or even the overall economy (if a societal perspective is chosen). It is not uncommon that a new intervention is cost effective only because costs are shifted to another—less expensive—sector. Consider, for example, a new type of drug that can be administered in a primary care setting while the existing treatment alternatives required hospital care. If the decision makers are responsible for allocating the primary health care budget only, they may act uneconomically if they approve coverage, even if the drug shows a favorable cost-effectiveness ratio from an overall health care system point of view.

### The situation in Austria

**Fig. 2:**
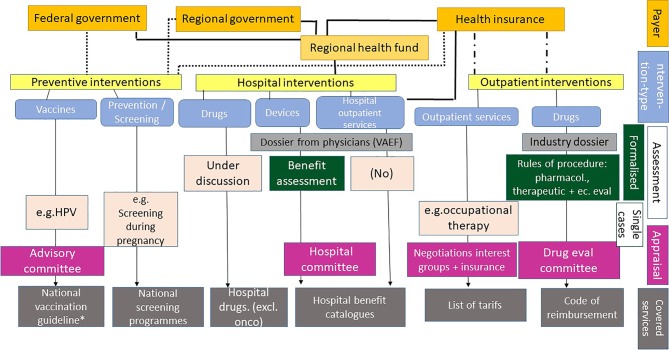
Benefit coverage processes in Austria. *VAEF* Verwaltung von Änderungs- und Ergänzungsvorschlägen für den Leistungskatalog, *HPV* human papilloma virus, *onco* oncology, *includes vaccines without public coverage

Austria has a social insurance-based health care system. Therefore, it can be classified as a Bismarck system according to the typology outlined above. However, in addition to health insurance—which funds 44% of health care expenditure—taxes and private sources play an important role, with a share of 30 and 26%, respectively. The overall health care spending in 2016 was 36.9 billion, representing 11.2% of the gross domestic product. Furthermore, the system is considerably fragmented in terms of governance and service provision. For example, the health insurance is responsible for outpatient services (including medication), while responsibility for hospitals (both hospital inpatient and outpatient settings) is mainly at the regional governance level. Responsibilities of preventive activities differ according to prevention type. Some rest within the federal government (e. g., vaccines), others within the health insurance (e. g., certain screening programs), but funding may be split between all of them (Fig. 2).

The role of health economic evaluations in pricing and reimbursement decisions differs across decision processes. Fig. 2 presents an overview on the different processes for coverage decisions. It shows that separate processes exist for pharmaceuticals and non-pharmaceuticals, and even within pharmaceuticals, processes are different between drugs paid by the health insurance for the outpatient sector and drugs used in hospitals. In none of the processes described do formal requirements to use economic evaluations exist, except for the decision-making process for outpatient pharmaceuticals, which falls under the responsibility of the Main Association of Austrian Social Security Institutions. In this process, the rules of procedure specify that each pharmaceutical product for which reimbursement is claimed needs to undergo economic evaluation. However, the term “economic evaluation” in this case primarily means executing price control based on the added clinical benefit of the new drug compared to existing pharmaceutical alternatives [25]. Economic evaluations as defined in this paper (CEA or CUA) are labelled “pharmacoeconomic studies” in the legal rules of procedure. They are required in two types of submissions: a) if the manufacturer claims that the drug offers a substantial additional benefit compared to existing drug treatment alternatives for all or a subgroup of patients for whom the drug is licensed for; and b) if the drug is submitted for listing in the “yellow box”^1^ of the code of reimbursement (*Erstattungskodex,* EKO^2^) and there are no alternative drug treatment options already listed [25]. The studies need to be submitted by the manufacturer as part of the dossier.

In the submission manual, basic reporting requirements for economic evaluations are outlined (title; research question; perspective; alternatives analyzed; type of economic evaluation; source of data used; quantitative results of patient benefits and costs; quantification of costs disaggregated by type of cost, quantities, and prices as well as direct and indirect costs; discounting; sensitivity analysis; summary of results) [25]. However, the manual does not specify methodological requirements, except for the types of costs that need to be taken into account (direct costs related to services paid by the health insurance, hospital costs, medical rehabilitation) and costs that are to be excluded (out-of-pocket payments). While methodological issues have partly been addressed in a consensus document initiated by a private industry consulting institute [26], no formal guideline specifying methodological details exists to date. Compared to many international guidelines, the document does not provide precise methodological guidance and leaves a lot of room for flexibility (e. g., concerning the outcome parameter used, the methods of sensitivity analysis applied, etc.) [27]. Its use is not mandatory and rather meant as a recommendation.

Little is known about the exact use of pharmacoeconomic studies in the internal processes and the role they play for the recommendations made by the drug evaluation committee. According to a recent report on drug reimbursement in Austria, no methodological handbook describing the methods that are applied for critically assessing the pharmacoeconomic studies and for summarizing the study results for the drug evaluation committee exists so far [5]. Both the reports for the drug evaluation committee as well as the recommendations the committee makes are confidential. The management of the health insurance makes the final decision on the inclusion into the EKO [25]. Few data are available on the role the pharmacoeconomic studies play in legitimating the final decision and/or in defining subgroups for which the drug may be publicly funded. A study from 2006 demonstrated that in almost all cases where pharmacoeconomic studies were part of the dossier, their use in decision making was rather restricted because of limited relevance and credibility of the study [28]. The degree of usage may have changed since then; however, systematic evaluations on the current use of economic evaluations in outpatient drug reimbursement processes are lacking.

In all other coverage processes described in Fig. 2, economic evaluations are used on a case-by-case basis only, if used at all. They have so far not played a role in defining the non-drug services covered by the health insurance in the outpatient sector. The situation is similar for coverage of services within the hospital, where different processes exist for hospital inpatient (separate for devices and drugs) and for hospital outpatient services. While benefit assessment of devices has gained considerable importance and has been increasingly standardized [29–31], cost-effectiveness evidence is not used in any steps of the process in a systematic way. One area where economic evaluations have played some role are coverage decisions for vaccination and for screening programs, although again, no standardized processes are in place. Three case studies demonstrate of how economic evaluations have been used in different coverage processes.

#### Case study TAVI

In 2017, the hospital providers from one Austrian region commissioned a systematic review of economic evaluations on transcatheter aortic valve implantation (TAVI) to an independent HTA body [32]. The reason for their request was to evaluate, retrospectively, whether their decision to restrict public reimbursement of TAVI to a specific sub-group of patients qualifying for TAVI according to clinical guidelines (high surgical risk caused by multimorbidity) would be supported by cost-effectiveness evidence. Among other issues, the analysis was triggered by international comparisons of TAVI use and reimbursement, which indicated that use was less restricted in other countries (e. g., Germany), resulting in much higher numbers of TAVI procedures and an increasing replacement of existing (and less costly) procedures. Within the systematic review, eight studies were rated as sufficiently relevant and of high quality. The results showed that TAVI compared to standard management (medical management in combination with balloon aortic valvuloplasty) was cost-effective in only two out of six analyses and resulted in controversial results compared to surgical aortic valve replacement. Sensitivity analyses of the included health economic evaluations showed that particularly the costs for treating serious complications (e. g., stroke) or the costs of the TAVI procedure had a significant impact on the cost-effectiveness results. The authors noted limitations in transferring the results to the Austrian context. While cost-effectiveness evidence was actively requested, currently, patients are selected based on clinical parameters such as severity of aortic stenosis, age, surgical risk, life expectancy, and comorbidities.

#### Case study HPV

In 2007, the Austrian Ministry of Health commissioned an economic evaluation of the human papilloma virus (HPV) vaccine. A CEA that compared vaccination of a) girls and b) girls and boys with screening for cervical cancer for a 52-year time horizon (2008 to 2060) both from a health care system and societal perspectives was conducted by an independent HTA body [33, 34]. The study applied a dynamic transmission model accounting for herd immunity. Effectiveness regarding life-years gained (LYG) of the vaccine was based on clinical trials (showing the reduction of HPV infections and precancerous lesions) and epidemiological data on the history of the disease from infection to invasive cervical cancer. Costs were based on Austrian cost data. The results showed that a 9 and 14% reduction in cervical carcinoma incidence in Austria would be expected by 2060 in the case of vaccinating girls only and girls and boys, respectively. The predicted reduction in mortality was 11 and 16%, respectively. The corresponding ICERs from a health care system were € 64,000/LYG when vaccinating girls only and € 311,000/LYG if girls and boys were vaccinated. The ICERs were lower when analyzed from a societal perspective. The results were most sensitive to the vaccine price and to the discount rate applied. The authors concluded that in the case of public coverage, the vaccine price should be considerably reduced, otherwise decision makers would run a high risk of high opportunity costs, thus losing more health elsewhere than that which was gained by the vaccine. As another less costly alternative, improvement of screening, was suggested. The vaccine was initially excluded from public coverage. Interviews among decision makers showed that they saw the study as a core reason for rejection [35]; however, the actual cost-effectiveness ratios were not part of the debate. On the contrary, it was the results on long-term cancer impact and the overall budget impact that received most attention. In 2013, reimbursement was approved for both girls and boys, albeit at a considerably lower vaccine price.

#### Case study diabetes

In 2015, a manufacturer claimed public coverage for a new drug for outpatient treatment of diabetes in Austria. The company argued that the drug has a substantial added benefit compared to existing treatment alternatives and was therefore obliged to submit a cost-effectiveness study as part of the dossier. According to the study authors, they conducted a CEA based on decision analytic modelling with the aim of analyzing the incremental costs per percentage of HbA1c^3^-decrease for a time horizon of 1 year from a health care system perspective. The effectiveness of the new drug was based on a manufacturer-sponsored head-to-head trial, which compared the new drug to insulin glargine and evaluated HbA1c as primary outcome. Costs were based on Austrian prices; however, only drug costs were included into the analysis. Univariate sensitivity analyses were applied to assess uncertainties. The study showed an ICER of € 3742/% HbA1c reduction and it varied between € 2139 and 14,942/% HbA1c reduction, depending on the level of Hb1c reduction assumed. In an additional analysis, the authors calculated how many cardiovascular events would be avoided by assuming a linear relationship between HbA1c level in their study and the reductions in cardiovascular events/mortality from other studies. The authors concluded that the drug is cost effective, the results are robust, and that the drug should therefore be included in the EKO at the price that was requested by the manufacturer. However, major flaws were identified in the critical appraisal of the study. The drug evaluation appraisal committee recommended inclusion of the drug into EKO based on the clinical benefit and on the final price–volume agreement that was negotiated.

## Discussion

### Challenges facing the use of economic evaluations in the Austrian system

When contrasting the Austrian system and the three case studies with the general challenges for using economic evaluation in the previous section (“Challenges to the application of economic evaluations”), the biggest limitation seems to be inherent in the Austrian health care system characteristics. Firstly, the Bismarck system means that—although health care resources are of course not infinite in Austria—the available budget is not as fixed in advance as it is the case in a Beveridge country such as the UK. However, as other cases with Bismarck-based systems demonstrate (Belgium, the Netherlands), a health insurance-based funding in itself does not seem to be the key challenge. It seems that rather the combination with other system characteristics that are inherent in the Austrian system such as fragmentation of funding, governance, and service provision play an important role. Not least, lack of transparency in the decision-making culture has been described as being a core barrier to the inclusion of (cost)-effectiveness evidence into the decision-making processes [24, 36]. For example, resource allocation decisions are often passed on to the level of the clinicians rather than addressing them at the macro-political level (especially in the inpatient sector [37]), which prevents the use of economic evaluation. Several initiatives have now started at the hospital-provider level to systematically evaluate high-cost hospital drugs before funding approval; however, CEA and CUA have so far not systematically integrated into the evaluation concepts [38]. In the case of outpatient drugs, where a form of positive list exists, according to the Austrian law, drugs that are excluded from the list can still be publicly reimbursed if approved by the chief medical officer (based on the individual needs of a patient). Thus, both the culture and the legal basis that form the Austrian health care system hinder a rational approach to resource allocation and thereby the use of economic evaluation. This does not mean that rationing is absent, but it rather indicates that rationing, which de facto always exists in a situation where demand exceeds supply, occurs implicitly.

The case studies demonstrate that efficiency generally has a low priority compared to other reimbursement criteria. As shown in the case of HPV vaccination, the ICER did not play a role in the discussions, while the burden of disease (cervical cancer incidence and mortality in Austria), the long-term impact on cancer epidemiology, safety issues, and the budget impact were all subjects of the political discourse [39, 40]. Even in the absence of a threshold, cost-effectiveness studies could be used to identify preferable patient subgroups. The final decision to publicly pay for vaccinating boys and girls despite a very high ICER demonstrates that this type of evidence is of low priority in justifying decisions. The case rather indicates that the health benefit, affordability, and prices are the core criteria for decision makers, rather than cost-effectiveness. This is also confirmed by the TAVI case, as it has been shown that decisions are based rather on clinical parameters alone, such as severity of aortic stenosis, age, surgical risk, life expectancy, and comorbidities, than on cost-effectiveness results. Efficiency seems at best to be used retrospectively to justify resource allocation decisions that were originally made based on affordability grounds or based on limited capacities that were required to restrict access to a manageable number of patients.

Other challenges outlined in “Challenges to the application of economic evaluations” also seem to be confirmed. Regarding applicability, data quality, and reporting/communication, members of the drug evaluation committee pointed out early that reasons for rejecting the use of economic evaluations were that the presentation of results was unsatisfying and difficult to understand. Furthermore, studies lacked transparency and the application of them to the decision problem was very limited because an inappropriate comparator was used or the study was not based on relevant Austrian data [28]. Some of these deficiencies still seem to exist. As the diabetes case study demonstrates, one reason for neglecting the study may have been its lack of transparency. Furthermore, the outcome parameter (€ per % of HbA1c reduction) and the time horizon (1 year despite the fact that diabetes is a chronic disease) used may likely have been meaningless for the decision problem, all of which resulted in low relevance for the Austrian decision context and low credibility of the study in general. Mayer et al. [17] showed that there are particular quality problems with cost data in Austria because of inconsistencies in the costing methods and cost-reporting standards. This poses an additional limitation to using reviews of economic evaluations (such as the TAVI case presented earlier), because it restricts the generally limited transferability of international study results even further.

Furthermore, the lack of methodological expertise and knowledge seems to be relevant in Austria. Members of the drug evaluation committee expressed concerns regarding their expertise in economic evaluations in a survey in 2006 [28]. Although more than 12 years have passed since this survey, none of the members of the drug evaluation committee is a (health) economist by training. The lack of knowledge is also visible in other decision processes, as an interview among members of the Austrian vaccination committee demonstrated [39]. The interviewee claimed that the HPV study demonstrated that the vaccination is not cost effective. However, this was mentioned nowhere in the report. In fact, in the absence of a cost-effectiveness threshold, a judgement on cost effectiveness based on the ICER is not even possible and has made interpretation of studies difficult (also demonstrated in the TAVI case). It seems to be common that economic terms are often used without a clear understanding of their meaning, which confirms the international findings outlined in “Challenges to the application of economic evaluations.”

### A potential way forward

The analysis has demonstrated that the challenges with using economic evaluations that have been identified in the international literature seem to also exist in Austria. It is therefore not surprising that economic evaluations have played a negligible role in Austrian coverage processes so far. Some of the challenges could be overcome quite easily if there is agreement among decision makers to systematically use economic evaluations as a source of evidence in reimbursement processes. For instance, education and capacity building will reduce the lack of knowledge among decision makers and increase the capacities of skilled staff involved in critical appraisal of industry studies, which, in turn, will reduce wrong perceptions and misunderstandings. Furthermore, standardization of methods and presentation of results as well as detailed quality criteria will increase the methodological quality of studies, their relevance for the decision problems, and the trust in the studies. To increase transparency, manufacturers could be obliged to submit not just the final study, but also the calculations the study is based on, as is the case in other countries (e. g., UK, the Netherlands, Finland, Sweden), where the decision analytic model has to be submitted alongside the study report. The methodological quality could be increased by a mandatory detailed guideline that clearly sets out the methodological standards and the reporting requirements for industry submissions, but also for economic studies that are commissioned to independent bodies. This shift towards more transparency of the studies would also reduce the perceived risks that economic evaluations may be misused. The submitted models may then be used by the decision makers themselves for analyzing different scenarios (e. g., different price scenarios).

However, the biggest barrier to integrating economic evaluations in reimbursement processes seems to lie within the nature of the health care system. On the one hand, the nature of the studies and the theoretical and ethical principles they are based on are in part incompatible with the Austrian legal context, the system characteristics, the moral values entrenched in the system, and the political culture. As a consequence, some of the currently existing methodological requirements for economic evaluation in Austria (e. g., to apply a health care system perspective and ignore out-of-pocket costs [25]) are in fact debatable within a Bismarck type of health care system. On the other hand, further neglecting of the notion of efficiency will threaten long-term sustainability of the health care system.

Copying how economic evaluations have been integrated into decision making elsewhere will likely fail, even if they refer to the same health care system type such as the Netherlands or Belgium, which are also rooted in the Bismarckian tradition [23, 41]. The situation rather calls for a process that takes the system characteristics into account. Increasing the use of economic evidence first of all requires an understanding among decision makers at all levels that addressing efficiency as one of a number of other decision criteria (and the rationalization and rationing issues that this brings with it) is not unethical, but that it may be rather unethical to ignore efficiency [23]. By passing on resource allocation decisions to clinicians, it remains nontransparent what type of criteria are applied and whether they reflect societal values (e. g., postcode rationing, allocation of resources based on health literacy, etc.). The value of CEA or CUA for decision makers in that sense is that it contributes to accountability by reassuring payers that their money is being spent wisely and by reassuring patients, caregivers, and the general public that their contributions to the health service are treated consistently [3]. The premise is, however, awareness that by using CEA or CUA, some discourse about willingness-to-pay based on a utilitarian philosophy would have to be introduced and that maximization of health would have to become one explicit out of several further decision-making criteria.

To tackle the applicability issue, a process needs to be initiated aiming at identifying what the objectives and needs of the decision makers precisely are [18, 22], i. e., what type of economic evidence they would find helpful and in what way this should be presented to facilitate its use. Both decision makers from various parts of the system and experts in economic evaluation and other forms of efficiency analysis would need to take part in this process. Existing reviews from other countries’ guidelines [42] and suggestions that have already been developed for Austria based on international examples [43] can serve as a starting point. But as the German case has shown [44], the process will also need to facilitate methodological discussions and it will need to be open towards adaptive and new forms of economic evaluations that may differ from the methods that are currently applied in other jurisdictions.

A starting point may be to draw on the suggestions from Williams and Bryan (2007) [11] that are to shift away from normative to more positive economics. Rather than a CEA or CUA, the primary type of economic evaluation to be used would then be a cost-consequence analysis that displays different effects and costs in a disaggregated way without combining results into a single indicator (such as an ICER). Instead of defining cost effectiveness based on willingness-to-pay thresholds in a normative (prescriptive) way, such studies describe the likely consequences in terms of costs and health which may be more approachable by decision makers [45, 46]. However, this needs to be weighed against the disadvantages of cost-consequence analysis: disaggregation results in limited comparability and generalizability; additionally, the absence of a single cost-effectiveness ratio may lead to less consistent decision making. Finally, leaving the weighing of the relative importance of different costs and benefits to decision makers bears the risk of cherry picking [45, 46]. Nevertheless, presenting costs and a range of outcomes in disaggregated form increases transparency, mitigates uncertainty, or makes more clearly visible what the core uncertainties are. Thereby, decision makers are supported with additional knowledge to be used to make informed decisions and to justify the decisions later on, including the value judgement they are based on. As the HPV case study has shown, the disaggregated presentation of knowledge may serve other purposes than just supporting efficient resource allocation (e. g., providing information on equity and affordability, requirements for program implementation, long-term health gains to be expected, etc.). Hence, it reduces the asymmetry of information that usually exists between payers and providers of interventions [47].

Regarding the process of conducting individual studies, the studies will likely be more relevant if decision makers and those who conduct the studies engage more actively in a communication process before the research starts, whereby the parameters to be addressed in the economic study are clarified (e. g., the type of comparator or the outcome measure used).

In parallel to discussions on efficiency and economic evaluation, a discussion on further attributes of benefit that may be valued alongside health gains (e. g., severity of a disease) needs to be initiated and criteria to represent those attributes need to be defined and made transparent.

## Conclusion

The increasing demand and high-priced technologies in health care have forced decision makers to think about how to spend resources wisely, and questions on efficiency have increasingly been on the political agenda in health care systems around the world. While it needs to be acknowledged that measuring efficiency is not a trivial undertaking, we argue that decision makers can no longer afford to ignore efficiency. Some countries have approached this challenge by systematically introducing CEA and CUA into coverage processes. In Austria, this has only happened to a very limited extent and if economic evaluations form a formal part of the evidence that is submitted for decision processes, such as in the reimbursement process for outpatient drugs, their role in justifying decisions or recommendations is currently unclear. Hence, the full potential of the studies in supporting consistent and transparent decisions seems currently not to be realized. This also includes the potential to use economic evaluation in other areas such as clinical guideline development. A number of reasons for this situation have been identified, whereby the biggest barrier seems to be that until now, methodological standards from countries that have rather different health care systems, legal contexts, and cultures have been suggested for Austria without taking into account the Austrian system context, the legal requirements that are the backdrop of specific societal values, and the needs of decision makers. Hence, a discourse is required on whether and how efficiency questions should be addressed in coverage processes. Most importantly, studies have to take the needs of decision makers into account better and may thereby challenge the current methodological discourse, without, however, sacrificing core methodological standards. One way forward could be to produce less prescriptive, but more descriptive economic evidence that simply adds further pieces of evidence, thereby fostering more evidence-informed decisions.

## Footnotes

1 The “yellow box” in the code of reimbursement summarises drugs that do not qualify for automatic reimbursement. Reimbursement is possible only after prior approval by the chief medical officer based on pre-defined conditions (e. g., restricted for specific subgroups of patients only).

2 The EKO is a positive list that includes all drugs that are publicly reimbursed if prescribed in the outpatient sector. However, drugs that are not included may also be reimbursed if approved by the chief medical officer; hence, a divergence from the core principle of a positive list exists in Austria.

3 HbA1c is a blood parameter which measures glycated hemoglobin and—if measured regularly—gives an overall picture of what the average blood sugar levels have been over a period of time. The higher the HbA1c level, the greater the risk of developing diabetes-related complications.
